# The role of health mediation in investigation of Hantavirus cases among informal settlements inhabitants of Cayenne area, French Guiana, 2022–2023

**DOI:** 10.3389/fpubh.2024.1364229

**Published:** 2024-06-25

**Authors:** Margot Oberlis, Marion Guyot, Paul Le Turnier, Luisiane Carvalho, Tiphanie Succo, Dominique Rousset, Benoit De Thoisy, Mélanie Gaillet, Anne Lavergne, Stéphanie Vandentorren, Loïc Epelboin

**Affiliations:** ^1^Équipe Mobile Santé Environnement, Croix-Rouge française, Cayenne, French Guiana; ^2^Santé publique France, Cayenne, French Guiana; ^3^Infectious and Tropical Diseases Unit, Centre Hospitalier de Cayenne, Cayenne, French Guiana; ^4^Centre d'Investigation Clinique Antilles-Guyane, Inserm 1424, Centre Hospitalier de Cayenne, Cayenne, French Guiana; ^5^Centre national de référence des Hantavirus, Institut Pasteur de la Guyane, Cayenne, French Guiana; ^6^Kwata NGO, Cayenne, French Guiana; ^7^Centres Délocalisés de Prévention et de Soins, Centre Hospitalier de Cayenne, Cayenne, French Guiana; ^8^Bordeaux University, Inserm, UMR1219, Vintage Team, Bordeaux, France; ^9^Santé publique France, Saint-Maurice, France

**Keywords:** investigation, Hantavirus, health mediation, socially vulnerable people, informal settlement

## Abstract

**Context:**

In 2022, four severe cases of Hantavirus pulmonary syndrome (HPS) were reported in patients from informal settlements around Cayenne, the main city in French Guiana. Regional Health Agency (RHA) was commissioned by the French Public Health Agency to estimate the seroprevalence of Hantavirus infections in the neighborhoods of confirmed cases of HPS. RHA then commissioned the French Red Cross (FRC) mobile public health team, providing support in environmental health issues to the population living in informal settlements by health mediators, to facilitate the investigation. The objective of this study was to describe the health mediators' activities set up to improve the efficiency of the investigation.

**Methods:**

The health mediators' team was specifically trained by virologist and infectiologist specialized in HPS. They helped the investigating team and health workers at various steps of the investigation. These interventions are then described in the results section.

**Results:**

The investigation took place between Nov. 2022 and March 2023 in three neighborhoods. During the pre-investigation activities, the mediators raised awareness about HPS of 343 people, among whom 319 (93%) planned to participate in the investigation. Altogether, 274 people finally participated in the investigation, including, i.e., 30.8% of the estimated population living in the three concerned settlements. The global proportion of patients with positive IgG anti-Hantavirus was 5.1%. The health mediators team supported the following steps: preliminary meetings and training modules, identification of resource persons, field visits and awareness and information campaigns (pre-investigation); on field data collection in informal settlements (per-investigation) and communication of individual results, public feedback meeting (post-investigation).

**Discussion/Conclusion:**

The involvement of mediators was probably a factor in the success of the public health response to socially vulnerable people living in the investigated neighborhoods. The preliminary prevention activities helped to raise awareness of the health risk and to enroll participants. Health mediation and outreach activities seem relevant tools of epidemiological field investigations in diseases affecting inhabitants of informal settlements.

## Background

French Guiana is a French overseas territory with an area of 83,846 km2 located in the North-East of South America, between Brazil to the East and South and Suriname to the West, covered by more than 90% of Amazon rainforest (1). This territory had a population of 301,099 at the beginning of 2023 (2). Its inhabitants are composed of different cultural populations including autochthonous populations (Amerindians and Maroons), Creoles (many descendants from French West Indies), Europeans, Chinese, Hmong, and from surrounding countries such as Brazil, Suriname, Guyana, but also, Haiti and Dominican Republic (1). The capital of the territory is Cayenne with an estimated population of more than 50,000 inhabitants. With more than half of the population living below the poverty line in 2018 and high demographic growth (+2.5% per year), the number of people living in precarious and isolated conditions is rising (3, 4). In a report published in 2022, the French Public Health Agency (*Santé publique France* – FPHA) reported that in France, the health status of the population was marked by a paradox: good health on average, but significant health inequalities (5). These health inequalities are even greater in the French overseas territories, particularly Mayotte and French Guiana (6). Social and territorial inequalities in health are glaring in this latter region. Obstacles to access to rights, prevention and care are numerous and affect a significant proportion of the population (7, 8).

Certain populations are affected by water-borne diseases in epidemic or endemic form, depending on the region. The geographical location of many villages in the interior and the administrative situation of informal settlements in coastal communities make access to drinking water and sanitation difficult. Whether in the dry or rainy season, the health risks associated with the lack of infrastructure and facilities persist. Epidemics of vector-borne diseases are cyclical (dengue, chikungunya, Zika viruses) and cases of leptospirosis are also frequently reported every year during the rainy season (9, 10).

This particular health situation is due to a number of specific factors, including a high frequency of non-connection to the public drinking water network (15% vs. 1% in mainland France) and a high proportion of informal settlements which represented 42% of settlements compared to legal settlements recorded in 2015 in the most densely populated communes, including 24% in potentially unhealthy areas due to lack of servicing and high population density (11, 12).

In November 2022, 65 informal settlements had been identified in coastal municipalities alone. Some 18,500 people were living there in extremely precarious conditions (13). Most of these people are isolated and have no legal status, with little access to health information and extremely restricted access to water, sewerage, or electricity networks. These factors make French Guiana an ideal location for deploying health mediation at all levels of care (primary, secondary and tertiary), and for integrating it into the healthcare system.

### Mediation in healthcare

Mediation emerged in France in the 1980s and 1990s, against a backdrop of socio-economic crisis and the deployment of interventions to help socially vulnerable groups. Initially social, then later health-related, mediation is now seen as a potential response to reduce social health inequalities (14–16). Mediation has emerged as a response to the needs in the field and was historically supported by civil society and then by institutions. This approach, which has been highlighted and officially recognized by public health policies since 2016, constitutes an instrument for modernizing the healthcare system by aiming to make it more effective in its fight against social inequalities in health (17, 18).

Defined by the French health authority as “the interface between vulnerable people who are far removed from the healthcare system and the professionals involved in their healthcare pathway,” health mediation is not just about communication or information (19). It relates to much broader issues of access to rights, care, and prevention, and involves “reaching out” to populations, professionals and institutions and “working with” individuals, with a view to empowering them (14, 18).

In France, access to social and healthcare rights for the entire population is theoretically guaranteed by law. However, major social and territorial health inequalities still exist. With the aim of combating exclusion and restoring equal access to prevention and care, health mediation is a response to these inequalities (16). Having demonstrated their usefulness, particularly with the most vulnerable populations, health mediators play a key role in intercultural contexts and with people living in precarious and isolated situations (18).

Health mediation is a particularly important approach in French Guiana, as it is considered essential in the fight against health inequalities (18). Deployed for some 20 years within the associative sector, it has developed considerably since the COVID-19 pandemic, providing support to vulnerable populations for whom problems of access to rights, care and prevention have considerably worsened.

Numerous programs, both associative and institutional, have integrated health mediation into their healthcare responses, aiming to reduce social inequalities in health by implementing healthcare responses that are as close as possible to the needs of these populations.

Concerned by major public health issues at regional and departmental level, the integration of this approach into health research projects and public health responses is also increasing (1). Aimed at improving the implementation and impact of interventions in specific contexts, this methodology provides an interface between target populations and all the stakeholders involved (20).

### Context of the intervention of health mediation in a public health response

Between March and September 2022, the National Hantavirus Reference Centre in French Guiana reported 4 confirmed cases of Hantavirus pulmonary syndrome (HPS) to FPHA and to the French Guiana Regional Health Agency (RHA). The reported patients resided in two communes of French Guiana, Rémire-Montjoly (2 cases) and Tonate-Macouria (2 cases). Among these patients, 3 had returned home and one died (21). HPS is a threatening disease linked to a virus of the *OrthoHantavirus* genus, transmitted through dust contaminated by the secretions of rodents (22). On this territory, only 7 cases had been reported since 2008 with a high lethality (23). Among them, two occurred in urban area (case in 2010 and 2020) and 5 in semi-rural environments compatible with the presence of wild rodents known to be reservoirs of Maripa strain infection (*Zygodontomys brevicauda et Oligoryzomys delicatus*). The lethality rate was high (4/7). However, the case in 2020 and the 4 cases in 2022 with a lower mortality (1/5), occurred in urban areas, more precisely in shanty towns on the outskirts of Cayenne, where these reservoirs are not usually found, but rather commensal rodents of the species *Rattus rattus, Rattus norvegicus* and *Mus musculus*. Rodent captures were carried out around the living quarters of the 2022 cases, and it was these latter rodents that were found, without the incriminating wild rodents being captured (21). One hypothesis put forward was that, in the context of environmental perturbations growing and unplanned urbanization, the virus had changed reservoir to urban rodents, but this could not be proven (24). Thus, the cluster cases of 2022 remain unexplained to this day.

A health public response through investigating on cases was therefore commissioned by the RHA to FPHA, with the main objective of estimating seroprevalence of recent and older Hantavirus infections in the inhabitants of the neighborhoods where confirmed cases of HPS lived in 2022. The secondary objectives were i/ to describe the characteristics of patients with positive Hantavirus serology when applicable, ii/ to analyze environmental and individual risk factors, iii/ to raise awareness among the target population of individual and collective preventive measures. French Guiana's RHA commissioned the French Red Cross (FRC) mobile public health team, providing support in environmental health issues to the population living in informal settlements by health mediators to facilitate the epidemiological investigation by FPHA and to provide individual and collective measures. The objective of this study was to describe and analyze the health mediators' interventions in (i) facilitating access to the field to the investigators and involving the target population in the project, (ii) raising the target population's awareness of individual and collective preventive measures against this infection.

## Methodology

### The methodology of the epidemiological investigation in brief

Clinical, behavioral, sociodemographic information, as well as the presence of HPS symptoms and risk factors, were collected from volunteers aged over 15 living in the survey areas using standardized questionnaires, as well as a blood sample. All data collection (questionnaires and blood sampling) was carried out by the FRC mobile public health team, supported by 2 nurses of the FRC trained for this specific intervention.

**Figure 1:**
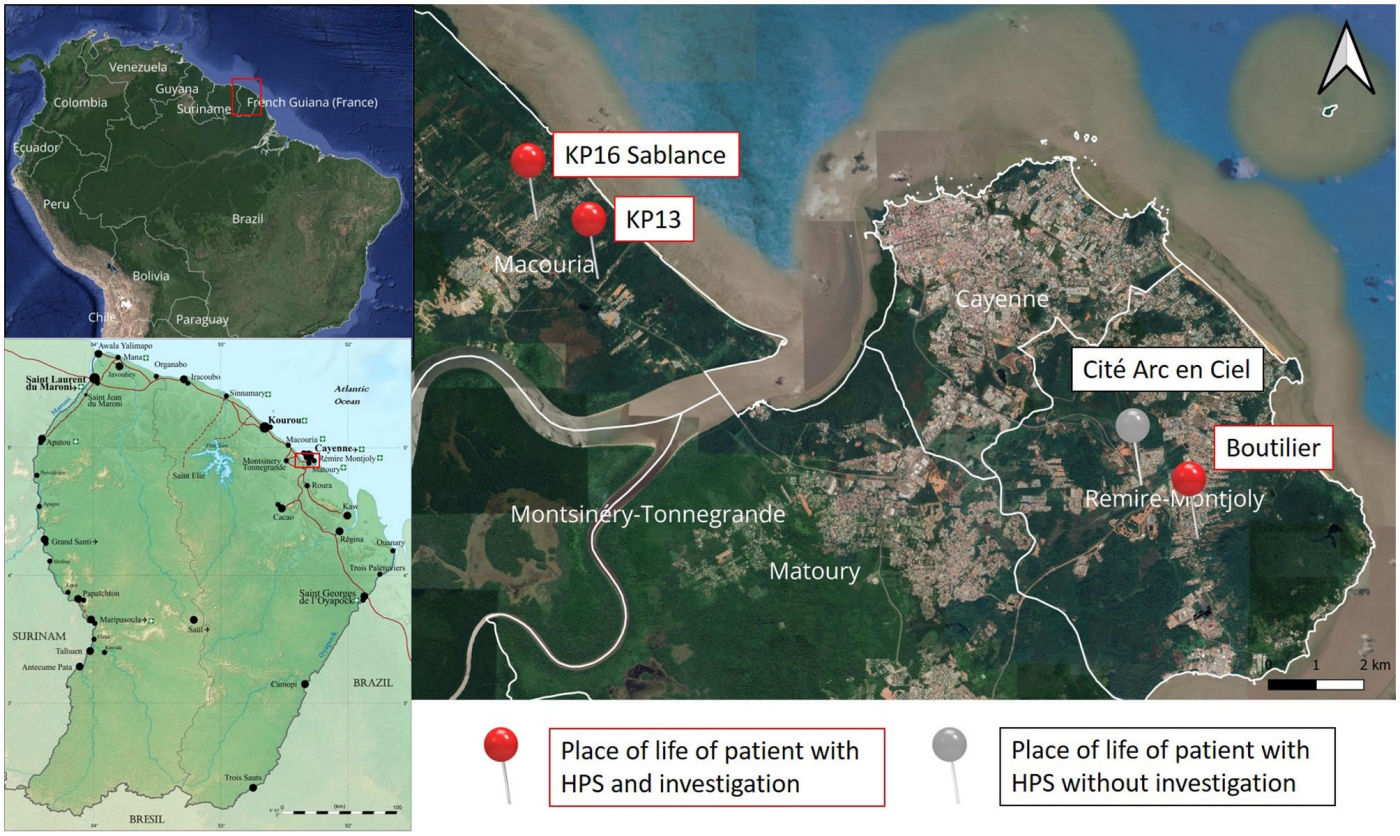
Map of French Guiana and location of the cases of Hantavirus Pulmonary Syndrome between January and September 2022 on a map of Cayenne and surroundings. KP: Kilometric point; HPS: Hantavirus pulmonary syndrome.

The investigation took place in the vicinity of three out of four confirmed cases of HPS diagnosed in 2022, in three informal settlements located in the communes of Macouria (*n* = 2) and Rémire-Montjoly (*n* = 1) (Figure 1). The fourth case lived in an isolated area. Therefore, no neighborhood investigation was considered, and this place was therefore excluded from the places of investigation.

As it is difficult to obtain reliable, up-to-date data in informal settlements, the size of the target population was estimated from a variety of sources: INSEE data on household structure in French Guiana, estimates based on satellite photos of the investigation sites, and estimates from the French Red Cross team who worked in these settlements. The size of the population to be surveyed was estimated at between 810 and 965 inhabitants in total, with an average number of 888 inhabitants. The investigation zones are described in the Table 1.

**Table 1:** Description and date of the investigations zones.

**Investigation zone**	**Sablance district (KP16)**	**KP13 district**	**Boutilier district**
Commune	Macouria	Macouria	Rémire-Montjoly
Occurrence of HPS case	September 2022	April 2022	March 2022
Estimated number of dwellings	220–270	25–45	NA
Estimated population over 15 year	486–597	56–100	268
Number of days of data collection	8	2	5
Number of days of reporting of results	2	1	2

Yo: years old; KP: Kilometric point; NA: not available.

### The FRC mobile public health team: from the WASH project to the mobile environmental health team

Created at the time of the COVID-19 pandemic, and funded by the RHA, the prefecture and European funds, the FRC mobile public health team, called “WASH project” (WAter, Hygiene and Sanitation), mainly made up of community health mediators (11 in total), worked with residents of informal settlements. Initially set up to limit the spread of COVID-19, the team covered almost the entire territory from September 2020 to May 2022. Its scope of action also expanded far beyond Covid-19, to include the prevention of vector-borne and water-borne diseases.

At the end of the health emergency, the team was resized and refocused on the informal settlements of the central coastal communities, to become the Mobile Environmental Health Team (MEHT). Funded by the RHA, the prefecture, municipalities, the team - made up of three health mediators and a project manager who is also a health professional - worked mainly on environmental health issues.

The team helped residents of informal settlements in and around Cayenne to reduce the health risks associated with their unhealthy environment (diarrheal, vector-borne and zoonotic diseases) by adopting health-promoting individual and collective behaviors. It encouraged community initiatives as part of a comprehensive, sustainable strategy to improve the living environment, and aimed to empower residents to take autonomous action in their neighborhoods (1, 25, 26). As a community-based health promotion project, the mobile team is mainly made up of mediators from the communities that make up the informal settlements in and around Cayenne. The team spoke several languages, enabling them to address the different communities they encountered: French, Guyanese Creole, Haitian Creole, English-speaking Guyanese Creole, Portuguese, Spanish and English, and had in-depth knowledge of the intervention context.

## Results

The MEHT was involved at various stages of the project in this public health response in partnership with healthcare professionals: pre-investigation, per-investigation, and post-investigation. The main stages in which the health mediators took part were team training and adaptation of tools, on-site visits, information and awareness campaigns, investigation in the neighborhoods, individual presentation of results in neighborhoods, and public presentations of results in the neighborhoods (Table 2).

**Table 2:** Different steps of the investigation in which health mediation had a role.

		**2022**								**2023**												
**Stage of the public health response**	**Step**	**S45**	**S46**	**S47**	**S48**	**S49**	**S50**	**S51**	**S52**	**S1**	**S2**	**S3**	**S4**	**S5**	**S6**	**S7**	**S8**	**S9**	**S10**	**S11**	**S12**	**S13**
Pre-investigation	Team training and adaptation of tools																					
	On-site visits																					
	Information and awareness campaigns																					
Per-investigation	Investigation in the neighborhoods																					
Post-investigation	Individual presentation of results in neighborhoods																					
	Public presentations of results in the neighborhoods																					

It should be noted that at the start of the survey, the FRC teams were supported by associative partners (DAAC, AAPSE) who intervened in the neighborhoods to reinforce the information and awareness campaigns. Not necessarily health mediators, they were responsible for informing residents about the investigation, while the FRC mediators were also responsible for raising awareness of the HPS.

### Quantitative results

**Figure 2:**
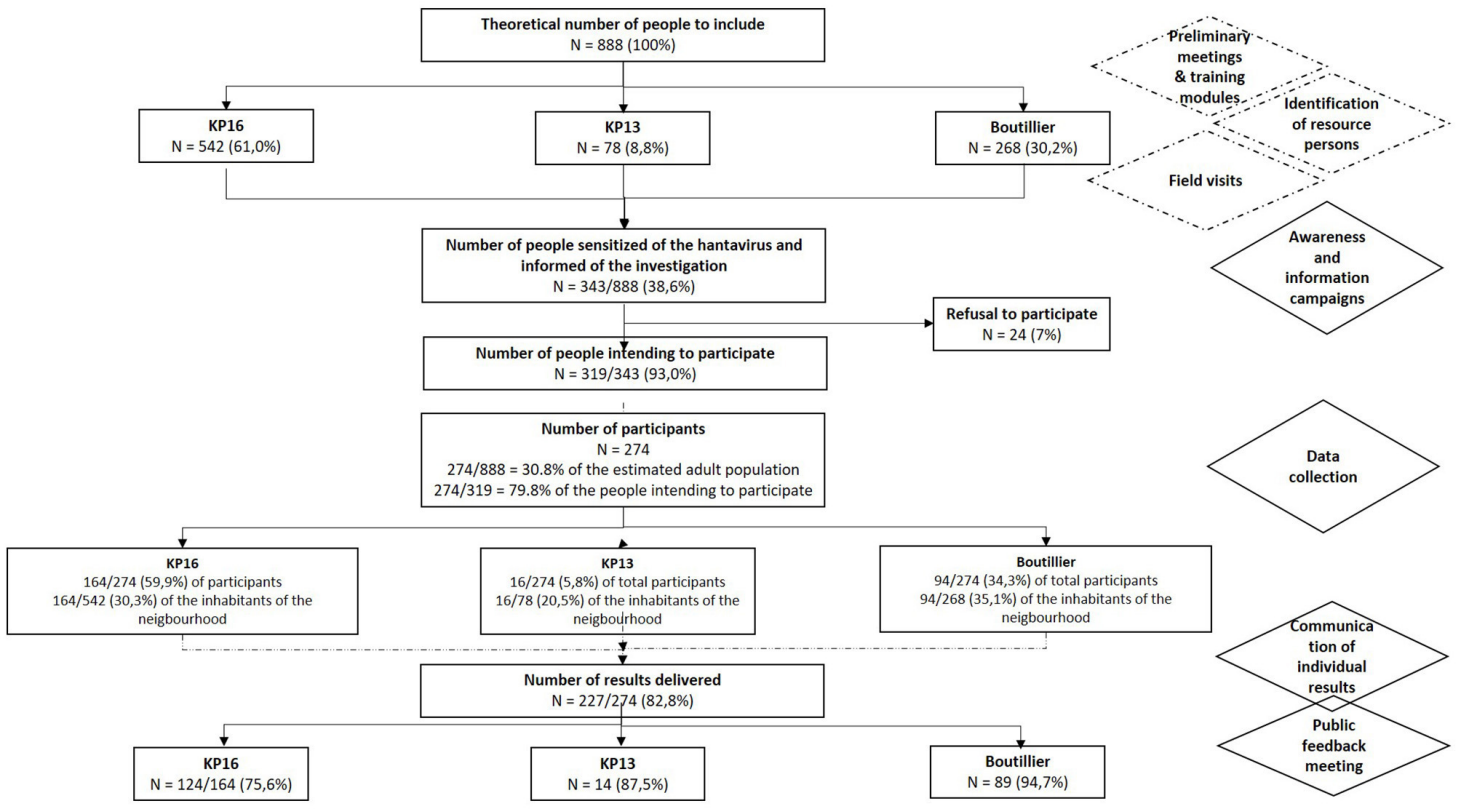
Flowchart of the investigation of HPS cases. KP, kilometric point.

The present publication focuses on detailing the intervention of health mediation in this public health survey, and not directly on the results of this survey. To briefly summarize the quantitative results of the investigation, among an estimation of 888 inhabitants older 15 years a total of 343 (38.6%) people was made aware of the HPS and were informed of the investigation's deployment during the outreach campaigns prior to the investigation (Figure 2). Among them, 319 (93.0%), intended to take part in the investigation after the awareness campaign. During 15 days, 274 people took part to the investigation, which corresponded to 30.8% of the estimated adult population and 79.8% of the number of people who intended to participate. The male to female sex ratio was 0.9, the median age was 42 years (IQR 25–75: 34–50, range 16–83) and 83% were born in Haiti. Results of the serological tests were communicated to 227 among 274 investigated persons (82.8%). The overall proportion of patients with positive serology was 5.1% (14/274), with a male to female sex ratio of 1.8, and therefore with a prevalence of 3.4% in women (5/145) and 7.0% in men (9/128).

## Pre-investigation

### Preliminary meetings and training modules

The RHA and FPHA organized several preliminary work sessions to bring together the institutional and associative stakeholders involved, to inform them in detail about the health situation, to present the Hantavirus investigation project and to adapt the investigation protocol, the data collection tools and the deployment of the intervention.

**Figure 3:**
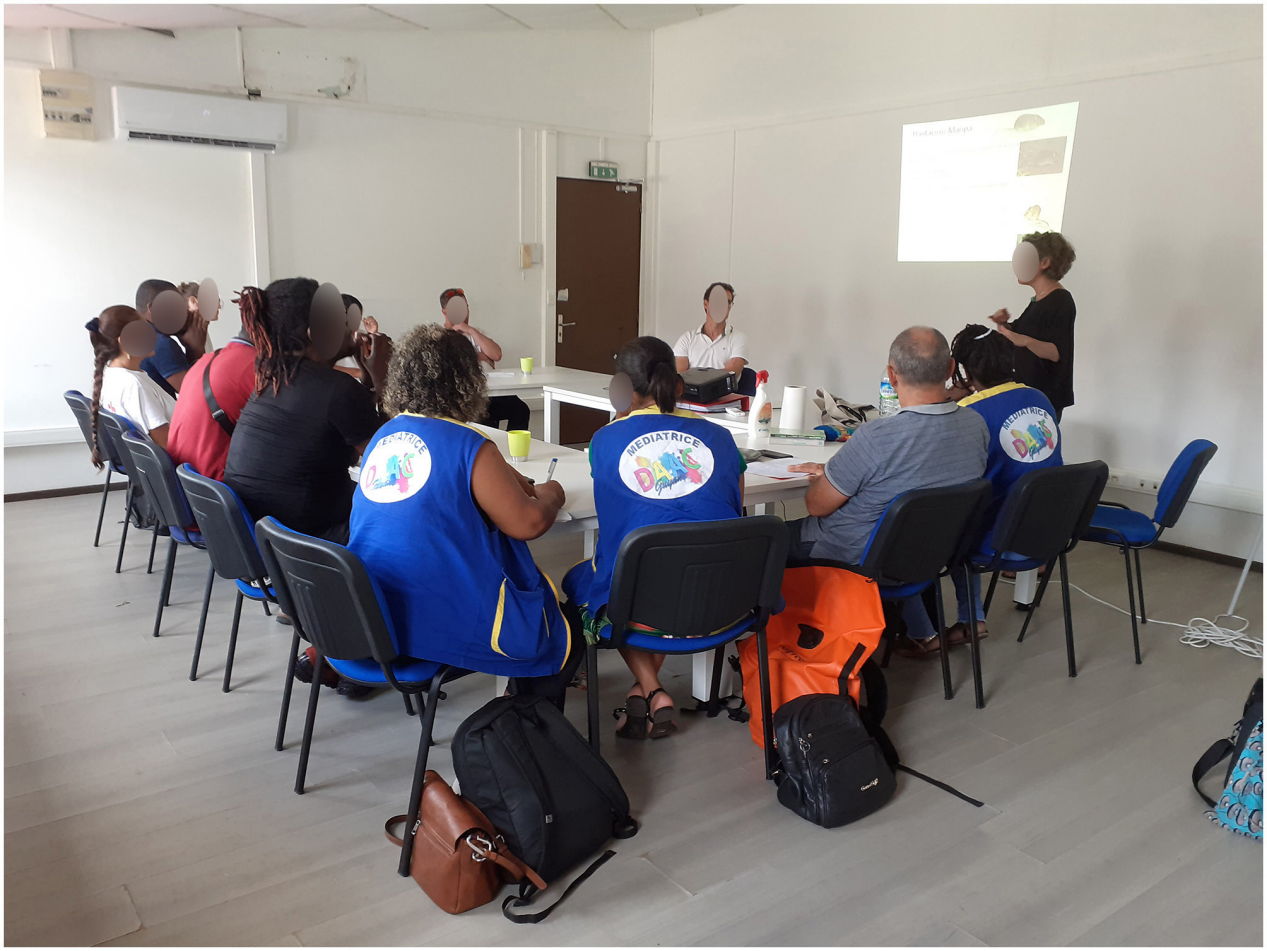
Training and discussions of the operational team (health mediators and nurses) with infectious diseases and virology specialists.

**Figure 4:**
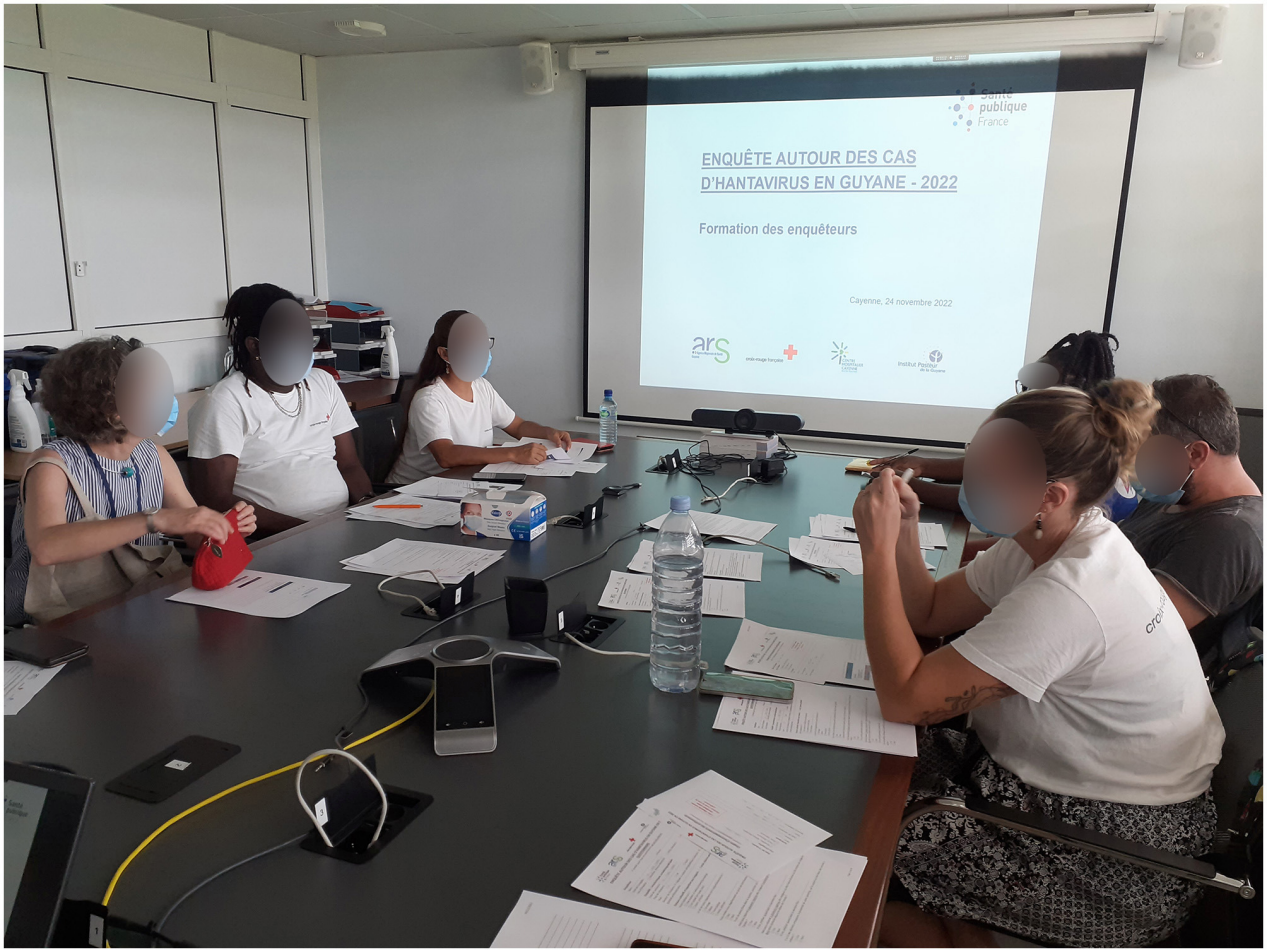
Training and discussions of the operational team (health mediators and nurses) with the professionals of the French Public Health Agency.

**Figure 5:**
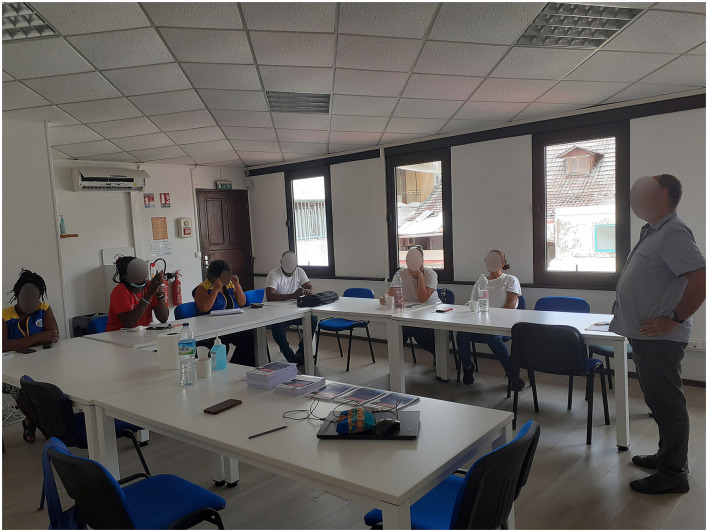
Training and discussions of the health mediators with the professionals of Guyane Promo Santé.

Three training modules were designed and delivered by different speakers to the operational team. A training course was designed to increase the knowledge on the HPS by members of the Infectious and Tropical Diseases Unit of Cayenne Hospital and the National Reference Center for Hantavirus of the Institut Pasteur in French Guiana presented in face-to-face meetings the virus, modes of transmission, main symptoms, evocative symptoms, and individual and collective prevention and protection measures (Figure 3). Then, training consisted in presenting the investigation protocol and methodology developed by FPHA to the health mediator team (Figure 4); this session enabled to finalize the tools based on feedback from the health mediators. Finally, a third practical training session was dedicated to the prevention postures and the development of health promotion messages, provided by the association “Guyane Promo Santé” (Regional Instance for Health Education and Promotion) (Figure 5).

Then, the role of the mediation team at this stage was to contribute to adapt the investigation tools to population. Indeed, the mediators' in-depth knowledge of the intervention areas and target audience enabled them to adapt and translate into several languages the various documents intended for participants (information note, investigation tools), to work on targeted information and prevention messages, and to anticipate certain difficulties at the time of the data collection phase by working upstream on their approach and posture.

### Identification of resource persons

To facilitate access to the field for the teams of investigators, and to encourage residents to take part in the investigation, resource persons were identified by the team of health mediators in each district. These reliable people, known and recognized by local communities, were either already known by health mediator team members due to previous actions in the neighborhoods, or they were identified during the field visits, specifically in the areas where the FPHA team located the investigation site. At this stage, the main role of the mediation team was to establish a bond of trust with the local population.

### On field visits

One or more site visits were made to each of the investigated neighborhoods, in partnership with the coordinators of the “Local Health Contracts” (LHC) of each concerned municipality. The LHC are a tool jointly developed by the regional health agency and a local authority to reduce territorial and social inequalities in health. It is the expression of local dynamics shared by actors and partners on the ground to implement actions, as close as possible to the population.

Carried out by the team of mediators, these visits made it possible to meet the resource people already known, to identify new people with a facilitating role in the implementation of the investigation (communication with residents, provision of a venue), to become acquainted with the investigation perimeters determined by FPHA, to draw up maps with landmarks that made sense to residents, and finally to identify places to welcome participants for the phases of data collection and restitution of individual results in the neighborhoods.

### Awareness and information campaigns

**Figure 6:**
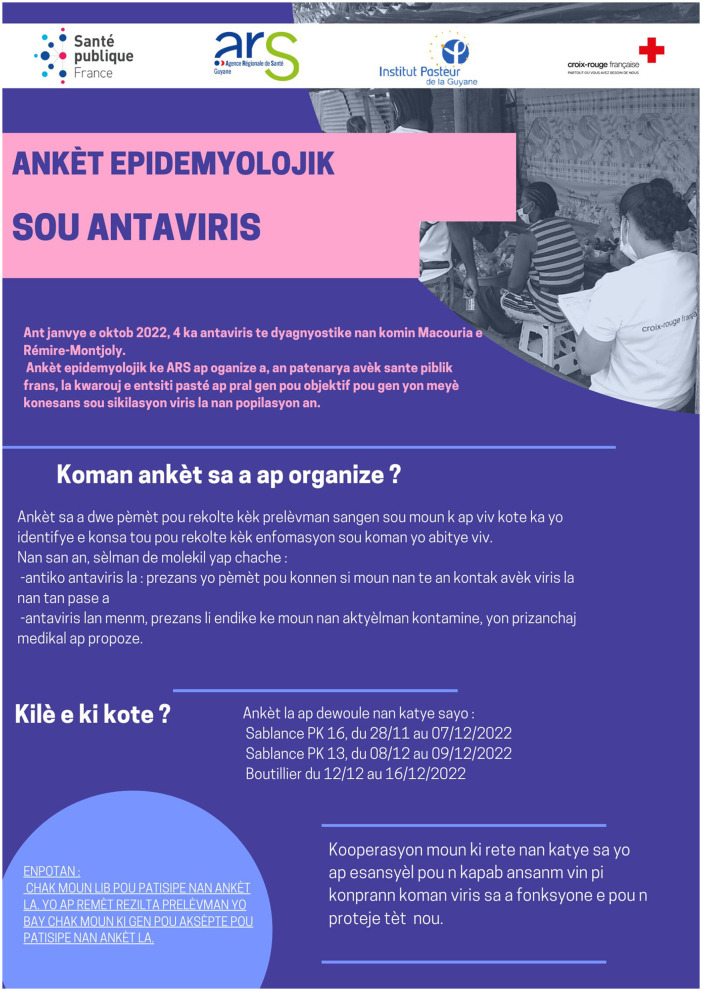
Written information in Haitian Creole to residents about the investigation.

**Figure 7:**
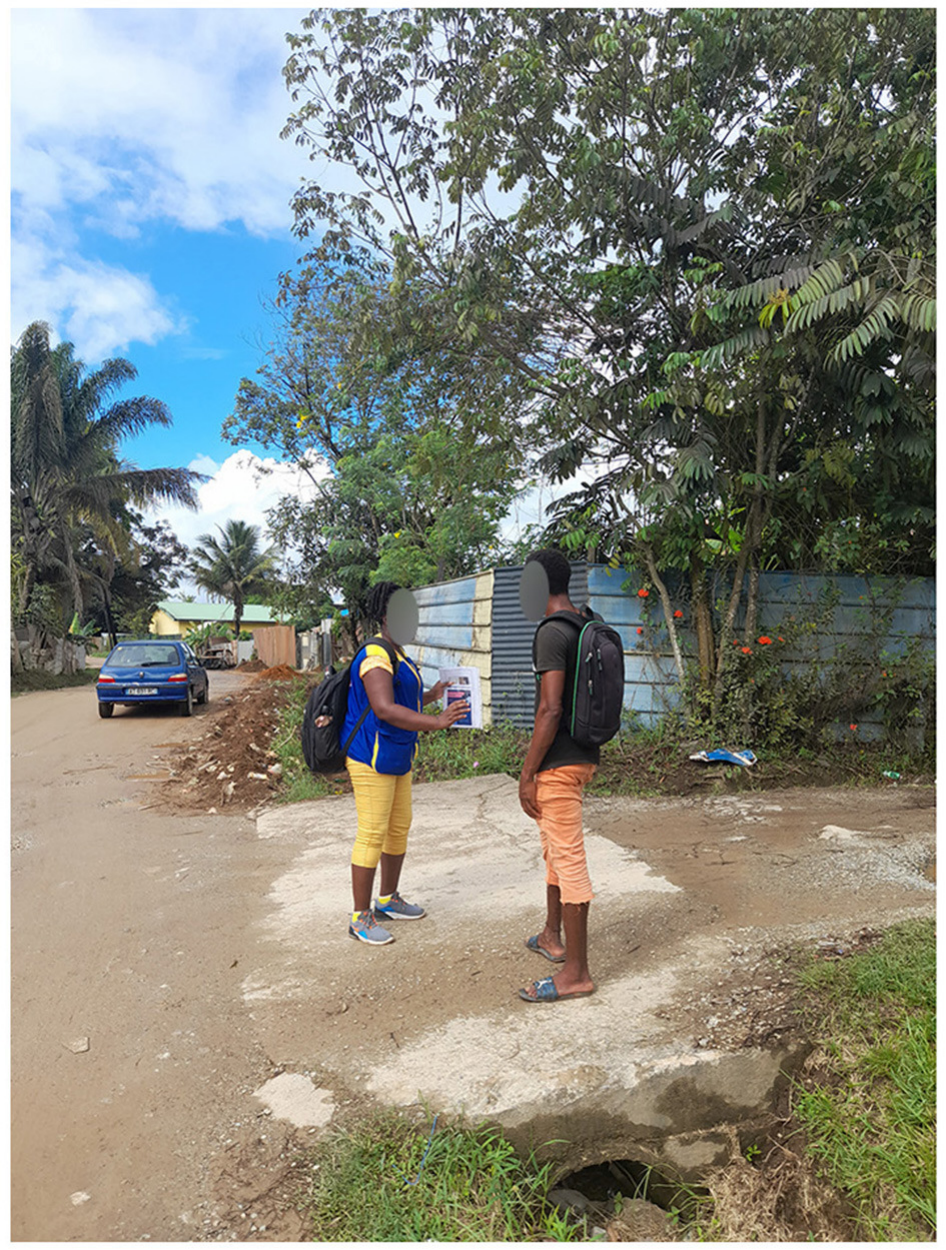
A health mediator raising awareness of Hantavirus Pulmonary Syndrome and informing a resident of an informal settlement about the investigation.

With the support of already-identified associative partners, the mediators carried out two awareness and information days in each neighborhood prior to the data collection phase (Figures 6, 7). The first occurred 1 week before the start of the investigation, and the second, the day before. The awareness-raising framework was drawn up in collaboration with health mediators, following the various training courses they attended. This included the main messages relating to Hantavirus infection and prevention (Appendix 1). These main messages were the description of the infection due to Hantavirus and its transmission, the most common clinical signs, what to do in the event of evocative symptoms, and the individual and collective preventive measures (avoid all contact with rodent urine, droppings and saliva, avoid breathing contaminated dust and prevent rodents from multiplying in and around homes). At last, they inform them of how the investigation would be deployed in their neighborhood, by giving them oral information and a leaflet translated into their mother tongue.

Already identified by the inhabitants of these informal neighborhoods during repeated previous interventions on water-borne diseases during COVID-19 pandemic, a bond of trust was quickly established during Hantavirus awareness-raising walks. The health mediators crisscrossed the neighborhoods in pairs, going door-to-door and approaching people directly on the street. They took the time to talk to residents and answer their questions about the Hantavirus and the investigation process. In addition, several key people in the neighborhoods were identified as community relays. They helped disseminate information about the survey to residents in advance of the investigation (oral information and via social networks) and were involved in organizing the investigation (providing a location for the investigation and mobilizing residents on the day).

## Per-investigation

### On field data collection in informal settlements

The data collection phase was carried out by the FRC team. This involved filling in the consent form and questionnaire, taking a blood sample and providing the information needed to retrieve the results.

**Figure 8:**
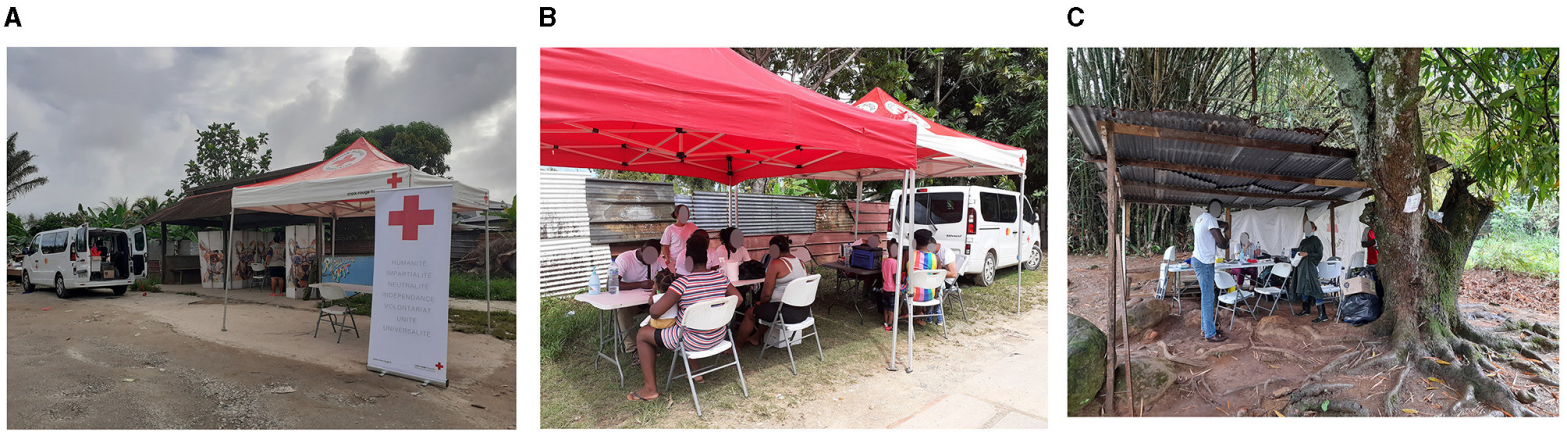
On field data collection in informal settlements in the three areas **(A)** KP16; **(B)** KP13; **(C)**. Boutilier. KP, kilometric point.

Divided into two teams, the health mediators visited local residents to inform them of the investigation team's presence, answer their questions about the investigation and raise awareness of the Hantavirus. The other team of mediators, working in pairs with the nurses, welcomed people at the sampling site, answered their questions, obtained their consent, translated and helped fill in the questionnaire, and interpreted for the nurses when necessary (Figures 8A–C).

## Post-investigation

### Communication of individual results

Once all the results were available, the health mediators contacted all the participants by telephone to inform them of the date and place of delivery, and to remind them of the procedure to follow to collect their results. The delivery of individual results was organized in partnership with the team from the Cayenne hospital's infectious and tropical diseases department.

**Figure 9:**
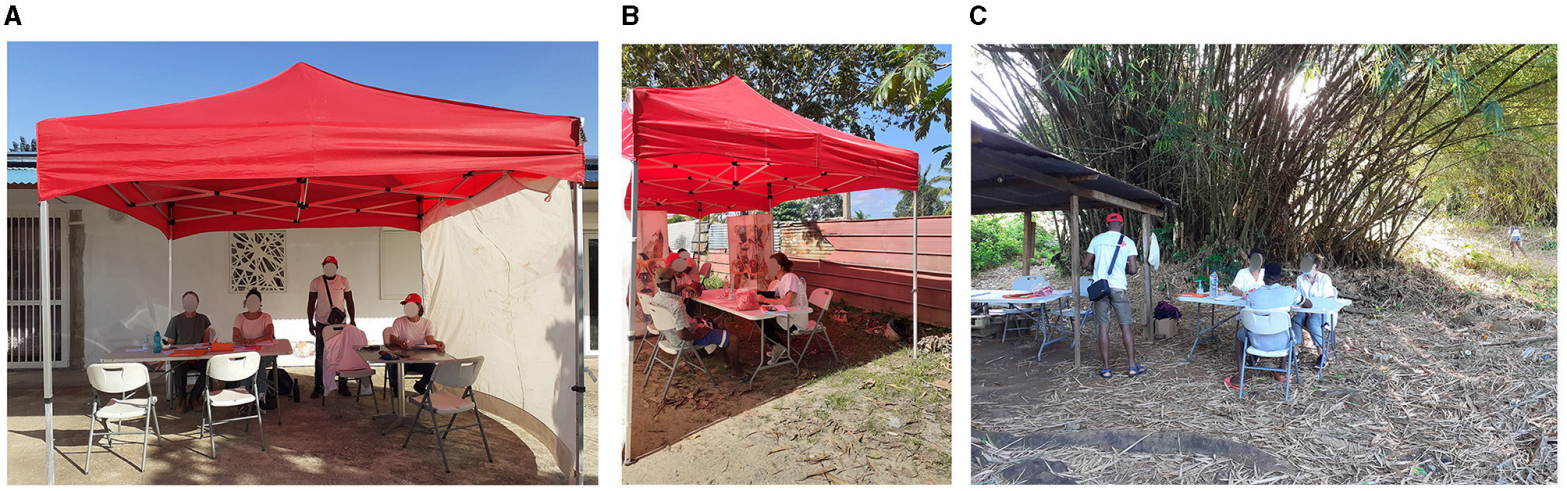
Communication of individual results in the three areas **(A)** KP16; **(B)** KP13; **(C)** Boutilier. KP, kilometric point.

On site, the mediators splitted in two teams. One was responsible for going out to the residents to inform them of the team's presence for the presentation of the results. The other team stayed on site in tandem with the infectious diseases physicians, to facilitate exchanges and provide interpreting if necessary and remind the inhabitants of prevention and protection measures besides physician's advice (Figures 9A–C).

### Public feedback meeting for collective results communication

**Figure 10:**
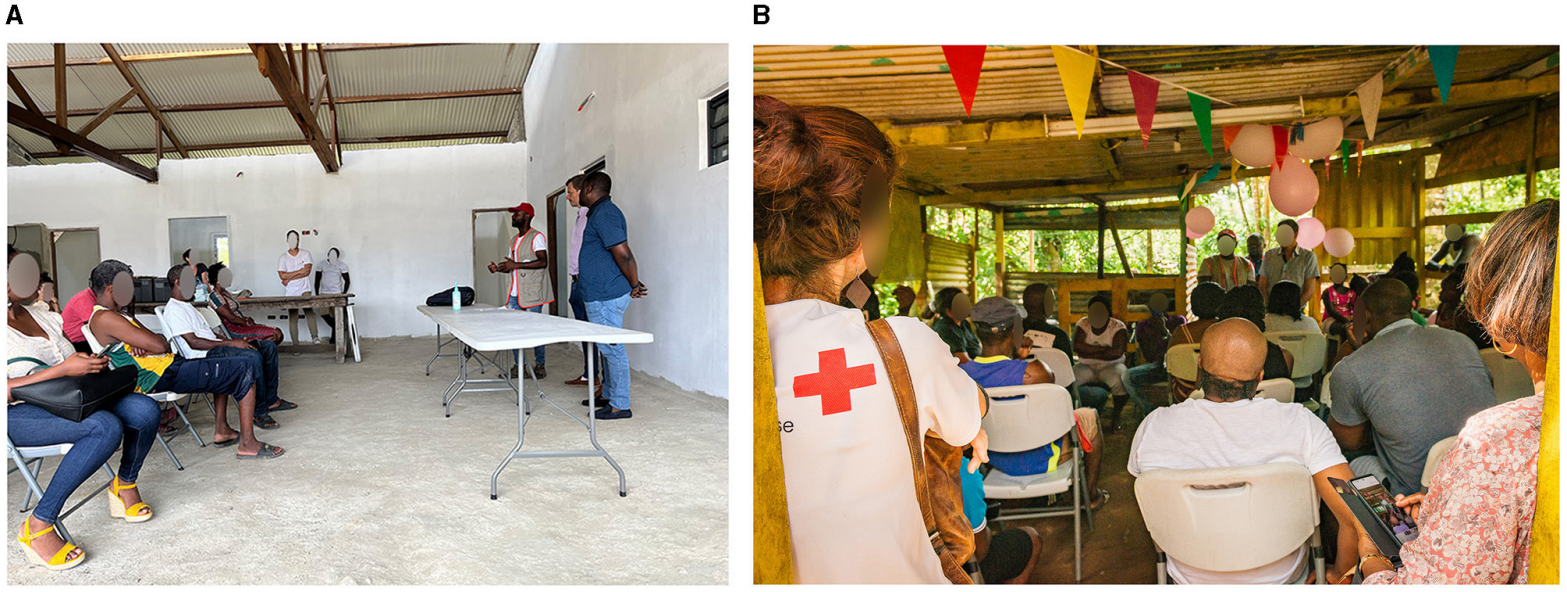
Public feedback meeting for collective results communication of two areas **(A)** KP16; **(B)** Boutilier. KP, kilometric point.

Two public feedback meetings were organized in Boutilier and KP16 districts to report on the main results of the investigation (seroprevalence in neighborhoods, main risk factors identified, profile of participants with positive Hantavirus serology, etc.), to remind people of individual and collective prevention measures, and to answer residents' questions about the investigation and the disease (Figures 10A, B). The health mediators organized these meetings, helped by identified resource persons to inform inhabitants. Information campaigns were carried out in advance by the mediators in each neighborhood, informing residents orally and displaying posters in the neighborhoods. These meetings brought together all the stakeholders involved in the public health response including institutions, partner associations, local authorities and local inhabitants. The health mediators welcomed the participants and provided interpreting services to help them understand the messages and facilitate exchanges.

## Discussion

This report highlights how health mediation took part in a local public health response with an intervention at each step of the process. Although there were no control groups or neighborhoods where the investigation was carried out without the help of health mediators, thus making it possible to estimate the contribution of health mediators to the success of the investigation, the participation rate at each stage seems satisfactory. Nearly 40% of this hard-to-reach population has been reached and sensitized, of whom nearly 80% participated in the investigation, of whom more than 80% came to collect their results. Ultimately, the results of the seroprevalence survey were based on 30% of the estimated population of these 3 informal settlements, which means that the results can be considered an accurate reflection of the prevalence in this population. For example, the most recent seroprevalence study carried out in French Guiana on arboviruses, Epiarbo, involved some 2,700 individuals sampled throughout the country, i.e., <1% of the population (27).

The intervention of the health mediators was reported to be decisive at each stage of this study to facilitate, make effective the participation and the return of the results to the residents of these informal settlements and play a key role in coordinating regional action. The design of this intervention brought together most of the theoretical conditions for the feasibility and success of health mediation, whether contextual (political and financial commitment), interventional [principle of otherness, interface function, intersectoriality function, mediation activities (going toward and bringing toward)] or specifically linked to the characteristics of health mediators (benevolence, congruence, ability to listen, posture favoring trust, non-judgment and sharing, strong collaborative activities between health mediators, local actors and the population) (17).

The involvement of the mediators have been a factor of success in the design of the tools, their use with the respondents and ultimately in the adherence to the investigation around the cases.

The climate of confidence between the inhabitants and the health mediators existed before the investigation because they were already intervening in these informal settlements. In the same way, the pre-existing knowledge of resource people in informal settlements with hard-to-reach people thanks to previous community health actions also greatly facilitated the various actions.

Pre investigation activities may improve involving residents during investigation (26), inform them of the occurrence of cases near their homes and raise awareness of the health risk to which they are potentially exposed as well as individual and collective prevention measures to protect against Hantavirus. The prior information on the deployment of the investigation, provided orally and in written form, and translated into several languages helped to arouse interest among residents, and the involvement of resource persons contributed to the support of the people. Health mediation and awareness-raising appear to be critical to the success of epidemiological field investigations, particularly in informal settlements.

In addition to awareness-raising and screening actions carried out on an *ad-hoc* basis, the increased and long-term presence of health mediators makes it possible to maintain a link with the inhabitants of informal settlements. Their regular meetings allow them to access health information and participate in the reduction of health risks by supporting behavior change. Health mediation seems to be essential to the success of these public health initiatives, in particular to enable target populations to access health information and increase their level of health knowledge, so that they can actively contribute to the prevention of certain diseases at both individual and collective levels.

Working in the neighborhoods investigated on a permanent basis, health mediators from the FRC and partner associations have maintained links with residents beyond the public health investigation conducted in November 2023. The health mediators' regular presence has enabled them to continue raising awareness and discussing the Hantavirus, answering residents' questions and referring them to health professionals. In addition to the investigation, which took place over a given period of time and mobilized a number of stakeholders, the team of mediators was able to continue anchoring itself in its informal neighborhoods, with its residents and with resource persons, facilitating actions that have subsequently continued around themes more usually dealt with by this team, such as the prevention of vector-borne and water-borne diseases but also referrals to the health system and support in accessing health rights. This action also enabled the team to better interact with local players and institutions, anchoring these teams in continuity. It should be noted that no new cases of HPS have subsequently been reported, despite initial fears of an emergence of this virus. As a result, the health mediator teams returned to other themes

Health mediation is an essential tool in the current context of a territory such as French Guiana. It was first deployed at the beginning of the 2010s by the associative sector to help people access their rights and then began to make timid inroads into the institutional environment, particularly within the hospital system, with the creation of mediator posts in several hospital departments, in delocalized prevention and care centers located in remote communities in the region, and with the deployment of a mobile public health team in the community (28). The health and social crisis that occurred during the COVID-19 pandemic put health mediators on the front line, bringing their profession to the spotlight (29–31). The number of projects involving this practice has multiplied within the various structures and research projects, especially around struggle against malaria and HIV (32). Other large-scale projects in progress used a similar approach involve mediation, such as the “Parcours d'Haïti” project (ClinicalTrials.gov identifier: NCT05174234), which studies the life of Haitian migrants in French Guiana, or the DEPIPREC project (ClinicalTrials.gov identifier: NCT05814068), which focuses on hypertension screening by community outreach workers for vulnerable populations, or MaHeVi, a study that aimed to evaluate the prevalence of viral hepatitis in the remote areas of the Maroni River in French Guiana (33, 34). These interventions can be compared with those carried out in mainland France, such as the Makasi project, which consists in organizing and evaluating an innovative intervention set up in the street, in places where people pass through (train stations, metro exits, etc.) to help people from Sub-Saharan Africa or the Caribbean in precarious situations to find out about and use the social and health resources available, enabling them to regain their strengths and their ability to act (35, 36).

This work did not satisfactorily assess the impact of health mediation during the investigation of HPS cases. Indeed, it describes and contextualizes the deployment of health mediation as part of a public health response. Thus, the evolution of the population's knowledge and the impact on the target population's adherence to this investigation were not evaluated. Although this study does not provide indicators of the impact of mediation, it does provide interesting information on the added value of involving health mediators in the various stages of a public health investigation. One of the strengths of this work is that there are few reported examples of mediation in healthcare, particularly in the context of an investigation in response to an epidemic signal. It provides a detailed method and an example of concrete deployment.

## Conclusion

We report here on the intervention of a team experienced in health mediation during the investigation of cases of HPS in the informal settlements of Cayenne, a multicultural French territory in America in the Amazon area. We provide here a detailed account of the entire intervention process of these teams at the various stages of its implementation (pre/per/post investigation). This exploratory work has shown the different roles of mediation, and it would be interesting, however, to include a proper and systematic evaluation of the health mediation intervention in investigations of episodic situations (such as malaria, dengue, or other emerging infectious diseases), in multicultural environments and with socially vulnerable people, as was the case here in informal settlements.

## Data availability statement

The raw data supporting the conclusions of this article will be made available by the authors, without undue reservation.

## Ethics statement

The studies involving humans were approved by the French Public Health Agency ethics committee. The studies were conducted in accordance with the local legislation and institutional requirements. The participants provided their written informed consent to participate in this study. Written informed consent was obtained from the individual(s) for the publication of any potentially identifiable images or data included in this article.

## Author contributions

MO: Conceptualization, Formal analysis, Investigation, Methodology, Validation, Writing – original draft, Writing – review & editing. MaG: Investigation, Writing – review & editing. PL: Investigation, Writing – review & editing. LC: Conceptualization, Data curation, Investigation, Methodology, Software, Writing – review & editing. TS: Conceptualization, Data curation, Investigation, Methodology, Resources, Software, Validation, Writing – review & editing. DR: Investigation, Writing – review & editing. BDT: Writing – review & editing. MéG: Writing – review & editing. AL: Conceptualization, Formal analysis, Investigation, Methodology, Writing – review & editing. SV: Writing – review & editing. LE: Resources, Software, Supervision, Validation, Writing – original draft, Writing – review & editing.
