# Supplementary material for: The role of health mediation in investigation of Hantavirus cases among informal settlements inhabitants of Cayenne area, French Guiana, 2022–2023
Source: Front Public Health. 2024 Jun 25;12:1364229. doi: 10.3389/fpubh.2024.1364229 (PMC11232507; doi:10.3389/fpubh.2024.1364229)
Supplement: Supplementary file 1 [file Data_Sheet_1.docx]

**Appendix 1 : INDIVIDUAL AWARENESS-RAISING FRAME (MARAUDING) THEME: HANTAVIRUS**

| **MAIN OBJECTIVE** | Raise public awareness of the existence of the hantavirus and the importance of preventive measures to combat this disease. |
| --- | --- |

**INTRODUCTORY MESSAGE**"Hello, my name is... and this is my colleague..., we work for the French Red Cross.
 We're here today to talk to you and learn more about the lives and behaviors of people in your neighborhood. If you agree, we'd like to talk to you about an illness caused by a virus: the hantavirus. Several people have been sickened by this virus since the beginning of 2022, including 1 who lived in your neighborhood. This means that this virus is present or has been present around you, and we're here to learn from you and make you aware of this virus.
We're not here to judge you, and we won't ask you for any personal information such as your name; there will be no consequences for you or your family if you don't wish to take part. Do you agree to give us a few minutes?"

**Hantavirus**

**WHAT IS HANTAVIRUS?**

The hantavirus is a virus that causes a disease that

- is transmitted to humans by an infected animal = rodent
- does not affect children
- may be serious or even fatal in adults
- is partly avoidable thanks to protection measures and the participation of the population in ensuring compliance with good practices.

**HOW IS THE HANTAVIRUS TRANSMITTED?**

- Viruses transmitted by specific rodents that are not normally found in cities but are more common in savannahs - these are not "classic" rats or mice => show pictures
- Most often: the virus is transmitted by inhalation (=breathing) of dust contaminated by the urine, droppings or saliva of infected rodents.
- The virus is also transmitted by contact with the eyes, nose or mouth after touching surfaces contaminated by the urine, droppings or saliva of infected rodents.
- More rarely: food contaminated by urine - droppings - saliva, bite of contaminated rodent
- Hantavirus is not transmitted from one person to another, so it is not contagious between humans.

**WHAT ARE THE MOST COMMON SIGNS OF HANTAVIRUS INFECTION?**

- First signs appear between 1 and 3 weeks after infection
- Most frequent first signs = sudden onset
  - Fever, severe fatigue, headaches, muscle aches - aches and pains (thighs, hips, lower back), "feels like the flu".
  - Other signs in 1 person out of 2 = stomach ache, diarrhoea, nausea, vomiting, malaise.
- Signs that come later (4 to 10 days after the first signs) =
  - Persistent fever, severe fatigue, weight loss, lack of hunger
  - Respiratory signs (cough, shortness of breath, difficulty breathing, sensation of suffocation, feeling of heaviness in the chest)
  - Sudden worsening of respiratory signs, sometimes leading to death

**WHAT TO DO IF SIGNS APPEAR?**

- Seek prompt medical attention if
  - Fever + breathing difficulties
  - Fever + severe fatigue, weight loss, lack of hunger

Very important, especially if contact with rodents is known!
These signs may also be another illness requiring treatment = consulting a doctor can identify the illness, assess its severity and treat it. There is no vaccine or medication against the hantavirus present in French Guiana.

**HOW TO PROTECT YOURSELF AGAINST THE HANTAVIRUS?**

- **ACTION 1: AVOID ALL CONTACT WITH RODENT URINE, DROPPINGS AND SALIVA**
- Closing up houses to keep rodents out
- Clean and protect food and beverage storage containers
- Clean cans with soap and water before drinking
- Trapping and capturing rodents
- Never touch rodents with your bare hands and wash your hands after handling them.
- **ACTION 2: AVOID BREATHING CONTAMINATED DUST**
- Wear a surgical mask when cleaning in/around the house if rodents are present
- Wear goggles and gloves if possible
- Moisten the floor before sweeping to prevent dust from flying away
- Use bleach to disinfect
- Wash hands with soap and water after cleaning
- **ACTION 3: PREVENT RODENTS FROM MULTIPLYING IN AND AROUND HOMES**

Rodents multiply near homes where there is garbage. They are thus present in or around people's homes if they do not dispose of their waste.

- dispose of household waste in garbage cans: if there is no garbage can, it is preferable to dispose of garbage in a garbage bag or liner and then dispose of it in a nearby dumpster
- keep your surroundings clean (inside and outside the house)
- protect the home against rodent intrusion: use products or traps, plug holes where rodents enter the house
